# Conserved Inhibition of Neutrophil Extracellular Trap Release by Clinical *Candida albicans* Biofilms

**DOI:** 10.3390/jof3030049

**Published:** 2017-09-06

**Authors:** John F. Kernien, Chad J. Johnson, Jeniel E. Nett

**Affiliations:** 1 Department of Medicine, University of Wisconsin, Madison, WI 53706-1521, USA; kernien@wisc.edu (J.F.K.); cjohnson@medicine.wisc.edu (C.J.J.); 2 Department of Medical Microbiology and Immunology, University of Wisconsin, Madison, WI 53706-1521, USA

**Keywords:** *Candida*, biofilm, neutrophil, neutrophil extracellular trap, fungal, reactive oxygen species, filamentation, phagocyte

## Abstract

*Candida albicans* biofilms are difficult to eradicate due to their resistance to host defenses and antifungal drugs. Although neutrophils are the primary responder to *C. albicans* during invasive candidiasis, biofilms resist killing by neutrophils. Prior investigation, with the commonly used laboratory strain SC5314, linked this phenotype to the impaired release of neutrophil extracellular traps (NETs), which are structures of DNA, histones, and antimicrobial proteins involved in extracellular microbial killing. Considering the diversity of *C. albicans* biofilms, we examined the neutrophil response to a subset of clinical isolates forming biofilms with varying depths and architectures. Using fluorescent staining of DNA and scanning electron microscopy, we found that inhibition of NET release was conserved across the clinical isolates. However, the dampening of the production of reactive oxygen species (ROS) by neutrophils was strain-dependent, suggesting an uncoupling of ROS and NET inhibition. Our findings show that biofilms formed by clinical *C. albicans* isolates uniformly impair the release of NETs. Further investigation of this pathway may reveal novel approaches to augment immunity to *C. albicans* biofilm infections.

## 1. Introduction

*Candida albicans* is the most common hospital-acquired fungal pathogen and frequently adopts a biofilm lifestyle, growing in communities which are adherent to medical devices or mucosal surfaces [1,2,3]. These fungal biofilms withstand high concentrations of antifungal drugs and survive immune attack [1,4,5,6,7,8,9,10]. Neutrophils, essential immune cells for rapid control of invasive fungal infection, are crucial for combatting candidiasis [11,12,13,14,15,16]. However, *Candida* biofilms resist killing by neutrophils, with neutrophils demonstrating a five-fold greater capacity for killing non-biofilm, or planktonic *C. albicans* over those in a biofilm [7,17,18,19].

Upon encounter with planktonic *C. albicans*, neutrophils release extracellular traps (NETs) [20,21,22,23]. These web-like structures of DNA, along with histones and other antimicrobial proteins, exhibit antifungal and antibacterial activity [20,24]. In particular, NETs have been shown to be critical for control of *C. albicans* hyphae, which are too large to be ingested by phagocytosis [23]. While the production of NETs may seem to be an ideal method of combatting fungal biofilms given their aggregative form, neutrophils fail to release NETs upon encounter with biofilms formed by *C. albicans* [19]. This impairment of neutrophil function has been linked to production of a biofilm extracellular matrix and is thought to contribute to the resilience of biofilms to host defenses. In addition, biofilms inhibit the generation of ROS, a key signaling and effector response in neutrophils [17,19]. 

Prior investigation examining the neutrophil response to *C. albicans* biofilms utilized the filamentous laboratory strain SC5314 [19]. This isolate produces robust biofilms of protruding hyphal cells which are the first to be encountered by immune cells, and inner yeast cells which appear to be protected. However, considerable variability exists among clinical isolates of *C. albicans* with regard to their biofilm-forming capacity, degree of filamentation, and biofilm architecture [25,26,27,28]. We reasoned that biofilm morphology may impact the impairment of neutrophil function. Differences in immune recognition between *C. albicans* yeast and hyphal cells are well-established [29,30]. These morphotypes engage different neutrophil receptors, leading to variations in the generation of ROS and the induction of pro-inflammatory cytokines [25,26,27]. Here, we examine the neutrophil response to clinical isolates of *C. albicans* selected for their differences in biofilm-forming capacity, architecture, and degree of filamentation. We demonstrate that these phenotypically distinct biofilms uniformly impair the release of NETs. 

## 2. Materials and Methods 

### 2.1. Strains and Inoculum

*C. albicans* SC5314, NCPF 3153, 98-210, and 98-17 isolates were used in this study. The NCPF 3153 strain was obtained from the National Collection of Pathogenic Fungi (Salisbury, UK). Strains 98-210 and 98-17 are clinical isolates from patients with invasive candidiasis [28]. Speciation was confirmed for all strains by PCR, as previously described [29]. Isolates were stored in a 15% (*vol*/*vol*) glycerol stock at −80 °C and maintained on yeast extract-peptone-dextrose (YPD) medium plus uridine (1% yeast extract, 2% peptone, 2% dextrose, and 80 μg/mL uridine) prior to experiments. Cultures were propagated overnight in YPD + uridine at 30 °C on an orbital shaker at 200 RPM. To prepare planktonic cells, 1 mL of overnight culture was inoculated into 20 mL of fresh YPD + uridine and incubated at 30 °C on an orbital shaker at 200 RPM for 2 h. The cells were then washed twice with Dulbecco’s phosphate-buffered saline, or DPBS (-calcium and -magnesium), and counted by hemocytometer. For fluorescence assays measuring reactive oxygen species production and free DNA, 3 × 10^6^ planktonic cells/well were used, a burden similar to the SC5314 control strain biofilm as measured by an XTT assay.

### 2.2. Microfluidic Device Model and Imaging

To determine the degree of filamentation of the clinical isolates during planktonic assays, we incubated 4 µL of planktonic cells (at 3 × 10^6^ cells/mL) in microfluidic devices for 4 h at 37 °C in Roswell Park Memorial Institute (RPMI) with 2% heat-inactivated fetal bovine serum (FBS) and glutamine (0.3 mg/mL). Two fields of view of 300 × 300 pixels were selected at random for each strain, and the number of both hyphae-producing cells and total cells was determined. We used the percentage of total cells that produced hyphae or pseudohyphae as an indicator of filamentation. To examine the biofilm architecture and thickness, biofilms were grown in straight channels of microfluidic devices (Iuvo Microchannel 5250, Thermo Fisher Scientific, Waltham, MA, USA) [19]. Fibrinogen at 10 µg/mL in DPBS was added to the channels for 1 h prior to inoculation, and rinsed three times with RPMI-MOPS before loading 2 µL *C. albicans* at 1 × 10^6^ cells/mL. The plate was incubated for 1 h with the inoculum at room temperature on its vertical axis, to allow the cells to adhere. The plate was then incubated vertically for an additional 24 h at 37 °C and imaged. Images were obtained on an inverted microscope (Eclipse TE300, Nikon, Tokyo, Japan), charge-coupled device camera (CoolSNAP ES2, Photometrics, Tucson, AZ, USA), and MetaVue imaging software v6.2. 

### 2.3. Biofilm Formation 

For biofilm formation assays, ROS assays, and Sytox Green assays, biofilms were formed in 96-well plates, as previously described [19,30]. Briefly, overnight cultures were resuspended in RPMI-MOPS at a concentration of 1.5 × 10^6^ cells/mL, and 200 μL of the suspension was added to each well of a 96-well plate and incubated at 37 °C for 24 h. For biofilm formation assays, an XTT (2,3-Bis-(2-Methoxy-4-Nitro-5-Sulfophenyl)-2*H*-Tetrazolium-5-Carboxanilide) assay was used to compare the burden of biofilm among the strains [30]. 

### 2.4. Human Neutrophil Collection

Blood was obtained from donors who provided written informed consent through a protocol approved by the University of Wisconsin Internal Review Board. Negative antibody selection via the MACSxpress Neutrophil Isolation and MACSxpress Erythrocyte Depletion Kits (Miltenyi Biotec Inc., Auburn, CA, USA) was performed to purify primary human neutrophils. Neutrophils were resuspended in RPMI 1640 (no phenol red) supplemented with 2% FBS. For all experiments incubations were performed at 37 °C with 5% CO_2_.

### 2.5. Scanning Electron Microscopy

For scanning electron microscopy studies, biofilms were grown on coverslips, as previously described [19,31]. Briefly, *C. albicans* (1 × 10^6^ cells/mL in RPMI-MOPS) were applied to a poly-l-lysine -treated plastic coverslip (13 mm, Thermonax plastic for cell culture) at 30 °C for 30 min. Following the removal of non-adherent cells, fresh RPMI-MOPS was added and cultures were incubated for 24 h at 37 °C and washed in DPBS. For planktonic studies, 1.5 × 10^6^ *C. albicans* in DPBS were applied to the coverslip for 1 h. Neutrophils (5 × 10^5^ cells) were then added on the coverslips for 4 h, which were subsequently processed for scanning electron microscopy, as described previously [2]. In short, the samples were washed with DPBS and fixed overnight (4% formaldehyde and 1% glutaraldehyde in PBS). After fixation, the samples were washed with PBS, treated with 1% osmium tetroxide for 1 h, and then washed again with PBS. The samples were then dehydrated via a series of ethanol washes, followed immediately by critical point drying. Samples were placed on aluminum stubs and sputter-coated with 14 nm platinum. The samples were imaged at 3 kV by a LEO 1530 scanning electron microscope.

### 2.6. Measurement of ROS

An oxidative stress assay was utilized to measure neutrophil ROS production, as discussed previously [19]. Neutrophils were incubated with the fluorescent dye CMH(2)CFDA (Life Technologies, Eugene, OR, USA) in DPBS at room temperature for 10 min in the dark and added to biofilms or planktonic cells at 2 × 10^5^ neutrophils/well. Phorbol myristate acetate (PMA, Sigma, St. Louis, MO, USA) at 100 nM was added in a subset of experiments as a positive control. Fluorescence (excitation 495 nm/emission 527 nm) was measured every 30 min for 3 h. Background fluorescence was measured for each *C. albicans* condition without neutrophils and subtracted from total fluorescence values prior to data analysis.

### 2.7. Sytox Green Assays 

A Sytox Green assay was used to identify NET release in biofilms [23]. Neutrophils (2 × 10^5^/well) were added to biofilm or planktonic cells. PMA (100 nM) was added in a subset of experiments as a positive control. After incubating the neutrophils for 4 h at 37 °C with 5% CO_2_, Sytox Green (Life Technologies, Eugene, OR, USA) was added to a final concentration of 1 μM and an automated plate reader was used to measure fluorescence (excitation 500 nm/emission 528 nm). For each condition, fungal background fluorescence was subtracted from total fluorescence values prior to analysis. Fungal background fluorescence represented approximately 5–10% of the positive, PMA-induced neutrophil control.

### 2.8. Fluorescent Imaging

To visualize the free DNA present in neutrophil-planktonic co-cultures, neutrophils were pre-stained with Calcein AM (Thermo Fisher Scientific, Waltham, MA, USA) at 0.5 μg/mL in DPBS at room temperature for 10 min in the dark and added at 2 × 10^6^ cells/mL to 3 × 10^7^ planktonic cells in µ-Slide 8-well plates (ibidi, Martinsried, Germany) [19]. Fifteen minutes prior to imaging, propidium iodide was added at 3 µM. Fluorescent images were obtained (excitation 480 nm/emission 525 nm and excitation 565 nm/emission 620 nm) with a 20× objective on an inverted microscope (Nikon Eclipse TE300) equipped with a charge-coupled device camera (CoolSNAP ES2) and MetaVue imaging software v6.2. Images were processed using ImageJ. For citrullinated histone detection, we used anti-histone H4 (citrulline 3) antibody (Millipore, Billerica, MA, USA) as described previously [19]. Images were acquired using brightfield and fluorescent (excitation 565/emission 620) detectors with a 40× objective on an inverted microscope (TI2-E, Nikon, Tokyo, Japan) equipped with a sCMOS camera (Orca-Flash 4.0 LT+, Hamamatsu, Hamamatsu City, Japan) and NIC Element imaging software.

### 2.9. Statistics

Experiments were performed at least 3 times using neutrophils from different donors on different days. Statistical analyses were performed by ANOVA or two-tailed Student’s *t*-tests using Sigma Stat or Excel software. Differences of *p* < 0.05 were considered significant.

## 3. Results

### 3.1. Biofilms Produced by C. albicans Clinical Isolates Differ in Architecture and Thickness

**Figure 1:**
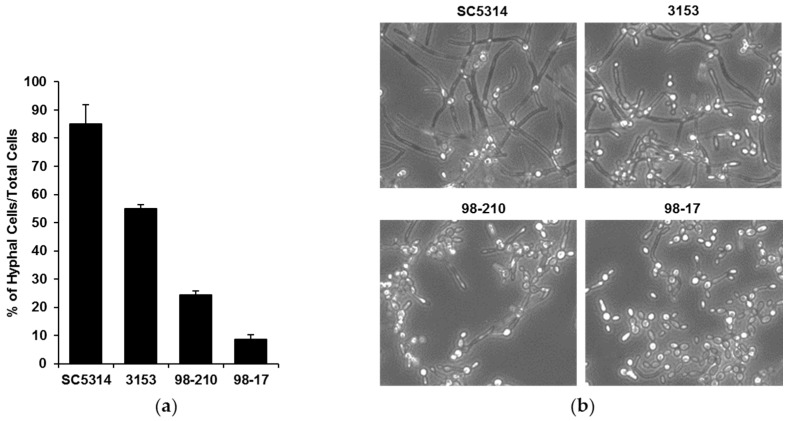
*C. albicans* clinical strains exhibit differing filamentation. (**a**) Filamentation of the clinical isolates was determined by incubating planktonic cells for 4 h in RPMI with 2% FBS at 37 °C in microfluidic devices and counting the cells producing hyphae as a percentage of total cells, SD shown; (**b**) Light microscopy images of clinical strains after 4 h of hyphal-inducing conditions (RPMI with 2% FBS at 37 °C, 20×).

**Figure 2:**
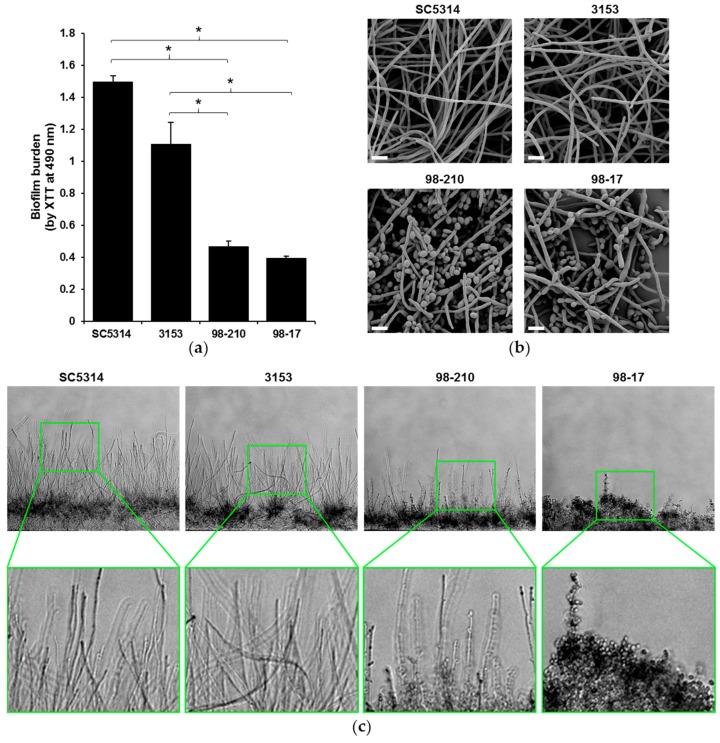
*C. albicans* clinical isolates have differing biofilm architectures. (**a**) The biofilm-forming capacity of isolates was estimated by an XTT metabolic assay after 24 h of biofilm growth. Statistical significance was analyzed by ANOVA with pairwise comparison by the Holm–Sidak method, * *p* < 0.05, *n* = 3, representative data with SD shown; (**b**) Biofilms were imaged by scanning electron microscopy to visualize the surface architecture. Measurement bars represent 10 µm for 2000× images; (**c**) The depth of the biofilms was examined by cross-sectional analysis of *C. albicans* biofilms growing in microfluidic devices. Original images were taken at 10×.

To investigate the influence of biofilm morphology on neutrophil function, we selected three strains of *C. albicans* to compare to the commonly used reference strain SC5314. The strains were selected for their varied degree of filamentation and biofilm-forming capacity. To assess filamentation, planktonic cultures were grown in the presence of RPMI with 2% FBS at 37 °C. After 4 h, the vast majority (85%) of the SC5314 cells had produced hyphae (Figure 1). The next most filamentous strain was 3153 (producing 55% hyphal cells), while the 98-210 and 98-17 cultures remained primarily as yeast forms, with only 24% and 9% hyphae, respectively. We next evaluated the capacity of the strains to form biofilms on polystyrene microtiter plates. Although each clinical strain formed a biofilm, the biofilms varied vastly in burden, as estimated using an XTT assay (Figure 2a). Strain 3153 formed a biofilm with a burden most similar to SC5314, reaching approximately 74% of the reference strain. In contrast, the biofilm burdens formed by the 98-210 and 98-17 isolates only reached 31% and 26% of the reference strain, respectively. The pattern of biofilm formation appeared to correlate with the degree of filamentation.

We used complementary microscopy techniques to examine the architecture of biofilms formed by the subset of clinical isolates (Figure 2b,c). To examine the outer surface of the biofilm, we grew biofilms on coverslips and imaged them using scanning electron microscopy (Figure 2b). Strain 3153 exhibited biofilm architecture similar to the SC5314 control strain, forming a dense biofilm with an outer layer composed nearly entirely of hyphal cells. A markedly lower degree of hyphal formation was observed in biofilms formed by strains 98-210 and 98-17, which both contained yeast forms on the biofilm surface. To further delineate the morphology of the biofilms, we utilized microfluidic devices, which permit cross-sectional examination of the biofilm architecture and depth [19]. The biofilms formed in microfluidic devices illustrated incredible differences in biofilm depth, which correlated with hyphal growth (Figure 2c). The strains displaying the highest degree of filamentation (SC5314 and 3153) also produced the thickest biofilms. In this model, strain 98-210 was moderately hyphal and 98-17 was composed almost entirely of yeast.

### 3.2. C. albicans Biofilms Variably Inhibit ROS Production by Neutrophils

**Figure 3:**
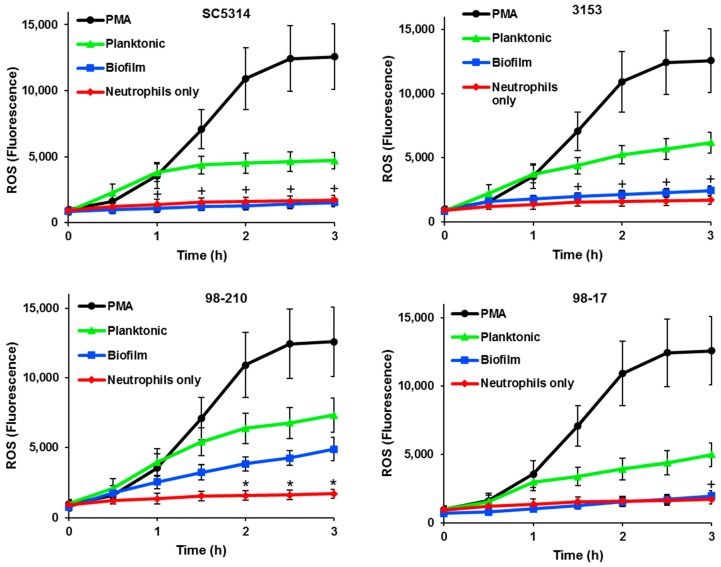
ROS production by neutrophils exposed to planktonic and biofilm *C. albicans*. ROS generation was estimated by fluorescence after neutrophils pre-stained with CMH(2)CFDA were co-cultured with clinical isolates. ROS production by neutrophils exposed to biofilms was compared to neutrophils only or planktonic cells at each time point using a Student’s *t*-test, * *p* < 0.05 biofilm vs. neutrophils only; ^+^ *p* < 0.05 biofilm vs. planktonic, *n* = 3, SEM shown.

Prior investigations have demonstrated the impairment of ROS production by neutrophils as a mechanism of immune evasion for biofilms [17,19]. These studies utilized the highly filamentous strain SC5314. We considered the possibility that ROS suppression may depend on biofilm architecture and examined ROS production in response to biofilms formed by the diverse subset of clinical isolates. We utilized CMH(2)CFDA, which permeates the cell membrane and fluoresces when it undergoes oxidation within the cell, allowing for the measurement of intracellular ROS. Like SC5314, each of the *C. albicans* clinical strains induced ROS production during planktonic growth, ultimately eliciting levels approximately 40–58% of PMA, an effective stimulator of ROS (Figure 3). Similar to the SC5134 control strain, biofilms formed by 3153 and 98-17 did not induce ROS production above the neutrophil alone control. Surprisingly, the 98-210 biofilm elicited ROS production significantly above the neutrophils alone control, reaching approximately 67% of the planktonic comparison. Factors underpinning this increase in ROS production uniquely for the 98-210 biofilm are unclear. While the biofilm formed by this strain contained more yeast morphotypes than either SC5314 or 3153, the biofilm with the highest abundance of yeast (98-17) fully inhibited ROS production. Combined, these data suggest that *C. albicans* biofilms do not uniformly suppress ROS production by neutrophils. Instead, the degree of this suppression likely varies by strain.

### 3.3. C. albicans Biofilms Uniformly Inhibit the Formation of NETs

**Figure 4:**
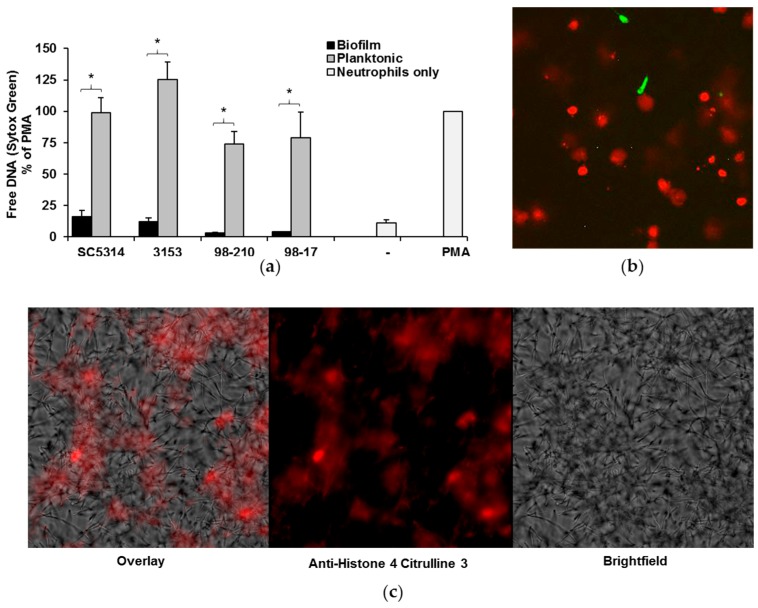
NET release by neutrophils exposed to *C. albicans* clinical strains. (**a**) Neutrophils were co-cultured with planktonic cells or biofilms for 4 h, and NET release was measured with a Sytox Green fluorescence assay. Neutrophils alone (-) served as a negative control, and neutrophils stimulated with PMA were included as a positive control and for normalization. The release of NETs upon encounter with biofilm and planktonic organisms for each strain was compared using a Student’s *t*-test, * *p* < 0.05, *n* = 5, SEM shown; (**b**) In order to visualize free DNA following neutrophil co-culture with planktonic *C. albicans*, neutrophils were pre-stained with Calcein AM (green) and co-incubated with planktonic cells (SC5314) for 4 h. After staining with propidium iodide (red), images were taken at 20×, and digitally enlarged 2× for comparison; (**c**) To assess the degree of histone citrullination, SC5314 planktonic co-cultures were stained with anti-H4 citrulline 3 antibody (red). Images were obtained at 40×.

Our next studies examined NET production in response to *C. albicans* biofilms. Previous investigation with SC5314 demonstrated impairment of NET production by biofilms and robust NET formation upon encounter with planktonic organisms [19]. To examine the influence of biofilm thickness and architecture on this inhibitory process, we utilized two complementary methods to measure NET production in response to our set of *C. albicans* isolates. The first assay utilized Sytox Green as a measure of free DNA and indicator of NET release. Results from multiple donors were normalized to PMA, a potent inducer of NETs, for comparison. Similar to SC5314, each of the isolates induced the formation of NETs during planktonic growth. By 4 h, NET production was approximately 74–125% of PMA control (Figure 4a). In stark contrast, minimal NET release was observed upon encounter with biofilms.

To further evaluate the elevated free DNA detected in planktonic co-cultures, we used fluorescent microscopy [32]. Staining with propidium iodide revealed the association of free DNA with neutrophils (red) (Figure 4b). Compared to the Calcein AM-labeled live cells (green), the propidium iodide labeled cells appeared enlarged, consistent with the release of NETs. We next evaluated these co-cultures by immunofluorescence and observed the presence of citrullinated histones, a hallmark of NET production (Figure 4c). The structure of the free DNA in the co-cultures, along with the strong presence of citrullinated histones, suggests that the majority of the free DNA we see in the planktonic co-cultures is a result of NET release.

**Figure 5:**
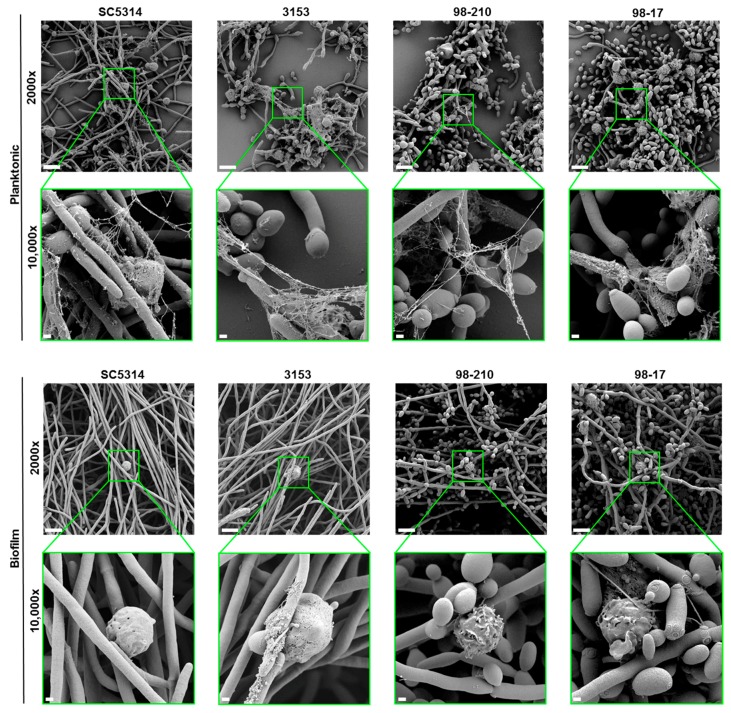
Scanning electron microscopy of neutrophils exposed to planktonic cells or biofilms of *C. albicans* clinical isolates for 4 h. Measurement bars represent 10 µm and 1 µm for images taken at 2000× and 10,000×, respectively.

As an additional assessment of NET release, we visualized Candida-neutrophil interactions by scanning electron microscopy (Figure 5). Consistent with the Sytox Green experiments, we observed NETs in response to planktonic cells for each of the *C. albicans* isolates. After a 4 h co-culture, thread-like webs coated groups of *C. albicans* cells. The fibers appeared to have an uneven thickness, potentially representing associated histones and other antimicrobial proteins. In contrast, each of the biofilms formed by these clinical strains failed to trigger NET release, as reported previously for SC5314 [19]. Taken together, these data suggest that biofilms formed by diverse clinical strains uniformly inhibit NET release, in contrast to their planktonic counterparts. This finding is interesting in light of the variable hyphal formation of these strains, suggesting that the hyphal architecture of biofilms is not crucial for inhibiting NET release.

## 4. Discussion

Invasive candidiasis is a widespread, life-threatening nosocomial infection [33]. Patients with *Candida* biofilm-infected medical devices are at particularly high risk for mortality [16]. Commonly used medical devices, such as vascular catheters, provide a surface for *C. albicans* to adhere and form resilient biofilms that are resistant to host defenses and conventional antifungal drugs [1,4,5,6,7,8,9,10,34]. Prior investigations utilizing a filamentous isolate of *C. albicans* linked biofilm immune evasion to the impairment of neutrophil function [19]. However, given the diversity of biofilms formed by *C. albicans* clinical isolates, we investigated the neutrophil response to a subset of clinical isolates which formed biofilms with variable thickness, fungal morphologies, and architecture. Our findings reveal that impairment of NET release is a mechanism to avoid immune attack that is conserved across diverse *C. albicans* biofilms.

The generation of NETs involves both ROS-dependent and ROS-independent pathways [35,36,37,38,39,40,41]. While previous study with SC5314 found biofilm inhibition of ROS, the current study shows this inhibition is strain-dependent for *C. albicans*. Unlike the other isolates, the biofilm formed by the 98-210 strain elicited the generation of ROS. The finding that this biofilm still impaired NET production indicates an uncoupling of the ROS and NET inhibitory pathways induced by *C. albicans* biofilms. Additional investigations are needed to further dissect these complex inhibitory pathways.

Biofilm formation by *C. albicans* frequently involves the production of long hyphae, which extend from the inner biofilm. As outer biofilm structures, these morphotypes would be expected to be encountered first by immune cells. Neutrophil-biofilm studies suggest a model of frustrated phagocytosis upon exposure to hyphal cells protruding from a biofilm [19]. However, here we show that even biofilms composed of predominantly yeast morphotypes are conserved in their ability to impair neutrophil function. The study shows that extensive hyphal formation is not required for this phenotype and supports a role for the involvement of other biofilm-specific components, such as the extracellular matrix.
